# Supplementary material for: Characterization of Zanthoxylum rhoifolium (Sapindales: Rutaceae) Essential Oil Nanospheres and Insecticidal Effects to Bemisia tabaci (Sternorrhyncha: Aleyrodidae)
Source: Plants (Basel). 2022 Apr 22;11(9):1135. doi: 10.3390/plants11091135 (PMC9101351; doi:10.3390/plants11091135)
Supplement: Supplementary file 1 [file plants-11-01135-s001.zip › plants-1672118-supplementary.pdf]

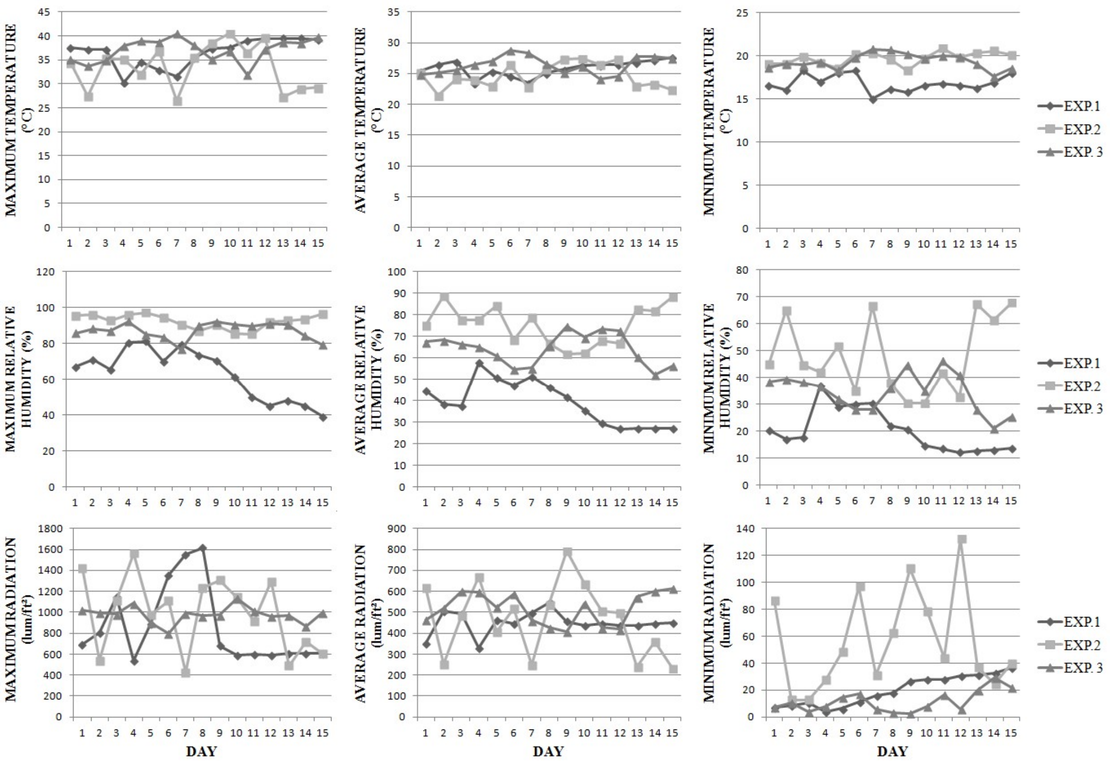

Figure S1. Temperature (°C), relative air humidity (%) and light radiation (lumens/ft²) recorded at screenhouse for experiments 1, 2 and 3.
